# Taxifolin increased semen quality of Duroc boars by improving gut microbes and blood metabolites

**DOI:** 10.3389/fmicb.2022.1020628

**Published:** 2022-10-14

**Authors:** Yexun Zhou, Liang Chen, Hui Han, Bohui Xiong, Ruqing Zhong, Yue Jiang, Lei Liu, Haiqing Sun, Jiajian Tan, Xiaowei Cheng, Martine Schroyen, Yang Gao, Yong Zhao, Hongfu Zhang

**Affiliations:** ^1^State Key Laboratory of Animal Nutrition, Institute of Animal Sciences, Chinese Academy of Agricultural Sciences, Beijing, China; ^2^Precision Livestock and Nutrition Unit, Gembloux Agro-Bio Tech, University of Liège, Gembloux, Belgium; ^3^YangXiang Joint Stock Company, Guigang, China; ^4^Yinuo Biopharmaceutical Co., Ltd, Harbin, China; ^5^College of Life Science, Baicheng Normal University, Baicheng, Jilin, China

**Keywords:** Taxifolin, semen quality, blood metabolite, gut microbiota, boar

## Abstract

Taxifolin (TAX), as a natural flavonoid, has been widely focused on due to its strong anti-oxidation, anti-inflammation, anti-virus, and even anti-tumor activity. However, the effect of TAX on semen quality was unknown. The purpose of this study was to analyze the beneficial influences of adding feed additive TAX to boar semen in terms of its quality and potential mechanisms. We discovered that TAX increased sperm motility significantly in Duroc boars by the elevation of the protein levels such as ZAG, PKA, CatSper, and p-ERK for sperm quality. TAX increased the blood concentration of testosterone derivatives, antioxidants such as melatonin and betaine, unsaturated fatty acids such as DHA, and beneficial amino acids such as proline. Conversely, TAX decreased 10 different kinds of bile acids in the plasma. Moreover, TAX increased “beneficial” microbes such as *Intestinimonas*, *Coprococcus*, *Butyrivibrio,* and *Clostridium_XlVa* at the Genus level. However, TAX reduced the “harmful” intestinal bacteria such as *Prevotella*, *Howardella*, *Mogibacterium,* and *Enterococcus*. There was a very close correlation between fecal microbes, plasma metabolites, and semen parameters by the spearman correlation analysis. Therefore, the data suggest that TAX increases the semen quality of Duroc boars by benefiting the gut microbes and blood metabolites. It is supposed that TAX could be used as a kind of feed additive to increase the semen quality of boars to enhance production performance.

## Introduction

The decreasing quality of semen is a serious issue that has contributed to a worldwide increase in infertility rates (10–15%) during the past few decades (Zhou et al., 2016; Wang et al., 2018). It is reported that semen quality (including sperm concentration and sperm motility) was reduced by about 50% worldwide between 1973 and 2011 (Centola et al., 2016; Levine et al., 2017). Environmental toxins, high fat diets, cancer treatments, and many other factors have been reported as involved in the rapid decline of semen quality (Vakalopoulos et al., 2015; Checa Vizcaíno et al., 2016; Skakkebaek et al., 2016; Virtanen et al., 2017; Han et al., 2019; Zhang et al., 2019; Ding et al., 2020). Many investigations have attempted to improve semen quality, and nutritional factors (protein, fatty acids, vitamins, and others) play crucial roles in semen quality. Lower protein or excessive protein can decrease sperm quality (Louis et al., 1994; Dong et al., 2016). Omega-3 (n-3) polyunsaturated fatty acids (PUFA), linolenic acid, eicosahexaenoic acid, and docosahexaenoic acid (DHA) can improve semen quality (Singh et al., 2021). Vitamins could also benefit spermatogenesis (Sanjo et al., 2020). Many dietary additives have been reported to regulate spermatogenesis and benefit semen quality. It has been reported that olive leaf extract, Korean red ginseng, and Genistein can improve spermatogenesis (Chi et al., 2013; Jung et al., 2015; Ganjalikhan Hakemi et al., 2019). Furthermore, we found that alginate oligosaccharides, beta-carotene, and chestnut polysaccharides improved spermatogenesis at various levels (Yu et al., 2020; Zhao et al., 2020; Ma et al., 2021).

Taxifolin (TAX) is a flavonoid present in a variety of plants, such as Douglas fir and fruits (grapes and oranges; Fukui, 1960; Topal et al., 2016). Due to its biological functions of anti-oxidation, anti-inflammation, anti-virus, anti-cardiovascular disease, and even anti-tumor activity, TAX has been used in food additives (in milk, cheese, and other foods), healthy products, and medicines (Galochkina et al., 2016a; Jomová et al., 2019; Turck et al., 2020; Zhang X. et al., 2020). TAX is a strong antioxidant that is mainly manifested in scavenging active oxygen and preventing the production of active oxygen (Jomová et al., 2019). It can alleviate LPS-induced acute lung injury, which triggers inflammation and apoptosis (Chen et al., 2017). TAX acts as an anti-fibrotic substance to effectively inhibit the fibrosis of the heart, kidney, liver, and lungs (Guo et al., 2015; Impellizzeri et al., 2015) *via* TGF-β/Smads and PI3K/AKT/mTOR pathways (Liu et al., 2021a). Moreover, TAX is an anti-viral molecule that inhibits Coxsackievirus B4 (Galochkina et al., 2016a, 2016b), and it can modulate the colorectal cancer cell cycle and apoptosis by regulating the Wnt/β-catenin signaling pathway (Razak et al., 2018). TAX has been found to have beneficial advantages for the reproductive systems. TAX could recover ovarian damage and reproductive dysfunctions through its antioxidant characteristics (Ince et al., 2020) and quite a few investigations have reported that it has beneficial effects on semen quality. The current research aimed to study the potentially positive effects of TAX on boar semen quality and the potential mechanisms involved to provide a basis for improving boar fertility. A few important proteins for sperm quality have been determined in the current study. The cation channel of sperm (CatSper), which is a kind of sperm calcium ion channel protein, plays a vital role in fertility *via* modification of the calcium entry and sperm hyperactivated motility (Lishko et al., 2012). Protein kinase A (PKA; the cyclic adenosine monophosphate (cAMP) dependent protein kinase) and ERK signaling have been reported to play important roles in sperm maturation, capacitation, and motility (Baro Graf et al., 2020; Li Q. et al., 2020). Zn-alpha2-glycoprotein (ZAG), *via* the cAMP/PKA signaling pathway, regulates sperm motility (Qu et al., 2007). Pigs have been used as an animal model to explore human nutrition because their physiology is to humans (O'Shea et al., 2015; Sun et al., 2021). In the current study, we discovered that TAX increased semen quality *via* the improvement of gut microbiota and systemic metabolome.

## Materials and methods

### Duroc boars and experimental design

**Figure 1:**
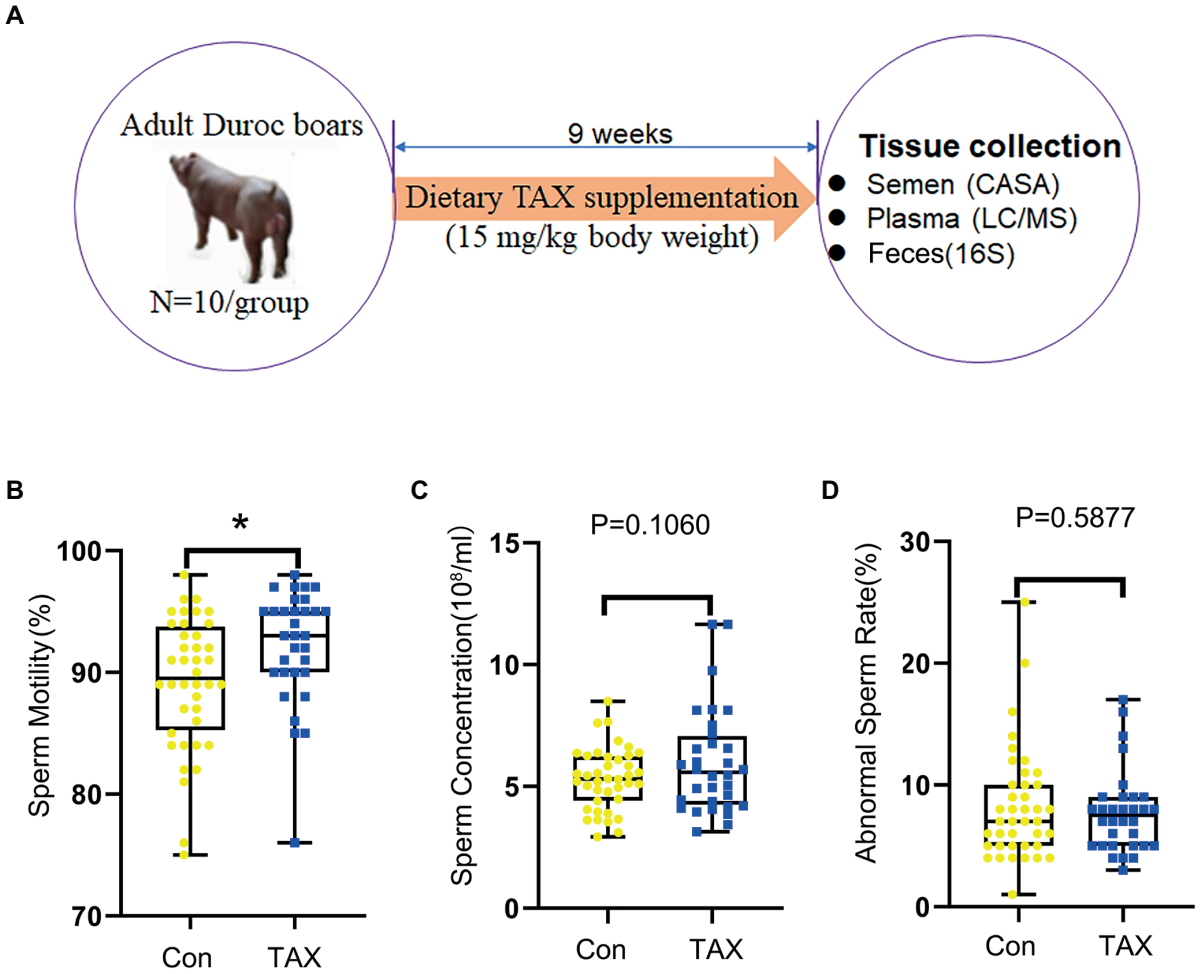
Effects of TAX on semen quality. **(A)** Study design. **(B)** Sperm motility. The y-axis represents the percentage of total cells. The x-axis represents the treatment (*n* = 10/group). ^*^p < 0.05. **(C)** Sperm concentration. The y-axis represents the concentration. The x-axis represents the treatment (*n* = 10/group). **(D)** Abnormal sperm rate. The y-axis represents the percentage of total cells. The x-axis represents the treatment. Data were expressed as the mean ± SEM.

The animal experiments were followed by the Animal Care and Use Committee of the Institute of Animal Sciences of the Chinese Academy of Agricultural Sciences (IAS2021-67). Twenty Duroc boars of similar age (2-year-old), health status, and weight (300 kg) were chosen along with a Tian Ti mountain boar stud from Yangxiang Joint Stock Company (Guigang, China; Guo et al., 2020). The boar feeding conditions we used have been previously reported by Wu et al. (2019). We divided these 20 Duroc boars into 2 groups randomly, each group included 10 boars in a control group (CON) and the Taxifolin group (TAX). The control group (CON) was fed a basal diet (Supplementary Table S1), and boars in the TAX group (TAX) were fed a basal diet with 15 mg/kg body weight TAX (Hou et al., 2021). TAX was provided by Yinuo Biopharmaceutical Co., Ltd., Harbin, China. The boars lived in individual pens and the whole feeding period was 63 days (Figure 1A).

The semen samples of Duroc boars were collected by a breeder who used gloved-hand technology. After that, sperm concentration, sperm motility, and abnormal sperm rate were assessed by CASA software according to the reported methods (Wu et al., 2019; Guo et al., 2020). Blood samples were taken from boar hind leg veins when they were working. We used anticoagulant tubes containing EDTA-2Na. Then, blood samples were centrifuged at 3000× *g* for 10 min to separate blood plasma, then transferred to a −80°C refrigerator until the experiments. Fecal samples were taken from the boar rectum, then placed in liquid nitrogen, and finally stored in a −80°C freezer for 16S analysis (Guo et al., 2020).

### Using computer-assisted sperm analysis system (CASA) to analyze semen quality of Duroc boars

The boar semen quality, including sperm concentration, sperm motility, and abnormal sperm rate were analyzed by a computer-assisted sperm analysis (CASA) system (Shanghai Kasu Biotechnology Co., Ltd., Shanghai, China; *n* = 10 per group; WHO, 2010; Zhao et al., 2016; Zhang et al., 2018, 2019). The evaluated criteria of sperm motility were as follows: grade A fast forward movement >22 μ m s^−1^; grade B forward movement <22 μ m s^−1^; grade C curve movement <5 μ m s^−1^; grade D none movement.

### Boar fecal microbiota sequencing

The protocol for the analysis of fecal microbiota was reported in our previous study (*n* = 10 per group; Zhang P. et al., 2020).

We used an E.Z.N.A.® Stool DNA Kit (Omega Bio-tek Inc., United States) to separate the total genomic DNA that came from the feces of the boars, according to the manufacturer’s instructions. A NanoDrop 2000 (Thermo Scientific, United States) and 1% agarose gel were used to detect the DNA quantity and quality, respectively. Primer pairs at 338F (5′- ACTCCTACGGGAGGCAGCAG-3′) and 806R (5’-GGACTACHVGGGTWTCTAAT-3′) amplified the V3–V4 region of the microbial 16S rRNA genes. The conditions of the PCR system and amplification were undertaken by following the technique used in our previous study (Wan et al., 2021). PCR amplification products can be extracted by 2% agarose gel and AxyPrep DNA Gel Extraction Kit (AXYGEN, New York, United States), which followed the instructions to purify them. After that, the sequences were assigned to the same operational taxonomic units ((OTUs) > 97% similarity).

### Plasma metabolome assay by LC-MS/MS

The plasma metabolites were detected as reported (*n* = 10 per group). Boar plasma was stored at −80°C. Firstly, the protein was removed from the samples and then analyzed by LC/MS using our previous research method (Zhang P. et al., 2020). Next, An ACQUITY UPLC BEH C18 column (1.7 μm, 2.1 × 100 mm) was employed in both positive and negative modes. Solvent A is an aqueous solution containing 0.1% formic acid. Solvent B is an aqueous solution containing 0.1% acetonitrile. The following program was: 5–20% B over 0–2 min; 20–60% B over 2–4 min; 60–100% B over 4–11 min. The composition was held at 100% B for 2 min, then 13–13.5 min, 100 to 5% B, and 13.5–14.5 min holding at 5% B. The flow rate was set at 0.4 ml/min and the column temperature was 45°C. The plasma samples were all kept at 4°C and the volume of the injection was 5 μl. ESI was used in the mass spectrometry program.

### Using immunofluorescence staining (IHF) to analyze the protein levels in boar sperm

The methods for IHF of boar sperm have been reported in our previous articles (*n* = 10 per group; Zhao et al., 2016; Zhang et al., 2019). Primarily, we fixed the boar sperm in 4% paraformaldehyde for 1 h, then air-dried the sperm, which was spread on slides covered with poly-L-lysine. After being performed 3 times (each time for 5 min) and then being washed with PBS, the sperm were incubated with 2% Triton X-100 in PBS for 1 h at room temperature. Next, they were washed 3 times (each time for 5 min) again with PBS, the sperm were blocked with PBS, which contained 1% BSA and 1% goat serum for 30 min at 17°C, and then incubated with diluted primary antibody (1:100; Supplementary Table S2) overnight at 4°C. The next morning, after being washed three times with Tween 20, the slides were combined with Alexa Fluor 546 goat anti-rabbit IgG (1,200) in the dark for 30 min at RT. The negative controls were only incubated with the secondary antibody. After washing the slides 3 times with the Tween-20, we then used DAPI (4.6-diamidino-2-phenylindole hydrochloride, 100 ng/ml) as a nuclear stain and incubated them for 5 min. After washing with ddH_2_O, we used a fade-resistant mounting medium (Vector, Burlingame, United States) to cover the slides. Therefore, the fluorescence images were obtained by the Microscope (LEICA TCS SP5 II, Germany).

### Quantitative detection of proteins by Western blotting

The procedure of Western blotting experiments, which are related to some beneficial sperm proteins, followed our previous publications (*n* = 6 per group; Zhao et al., 2016; Zhang et al., 2019). Sperm cells have to first be lysed in RIPA buffer that contains the protease inhibitor cocktail purchased from Sangong Biotech, Ltd. (Shanghai, China). Second, we detected the protein concentration followed by the instruction of BCA kits (Beyotime Institute of Biotechnology, Shanghai, China). In this study, Actin was used as a reference. The primary antibodies (Abs) are shown in Supplementary Table S2. The secondary donkey anti-goat and goat anti-rabbit was purchased from Beyotime Institute of Biotechnology (Shanghai, P.R. China) and Novex® by Life Technologies (United States) respectively. Next, we loaded 50 ug of total protein in each sample to 10% SDS polyacrylamide electrophoresis gels, which were transferred to a polyvinylidene fluoride (PVDF) membrane at the electric current of 300 mA for 2.5 h at 4°C. Then, we used 5% BSA to block the membranes for 1 h at 17°C, after washing them 3 times with 0.1% Tween-20 in TBS, the membranes were incubated with primary antibody, which was diluted at 1:500 in TBST with 1% BSA overnight at 4°C. The next day, Using TBST to wash three times, the blots were incubated with the secondary goat anti-rabbit or donkey anti-goat, respectively, for 1 h at 17°C. After being washed three times, the blots were imaged by a camera (Kodak, Beijing, China). Finally, we used ImageJ to analyze the bands.

### Statistical analysis

Data are expressed as the mean ± SEM. *p* < 0.05 was considered a significant difference. The student’s *t*-test (SPSS 21 software) was used to perform the statistical analyses. Spearman’s correlation analysis was completed by RStudio (version 4.0.3) platform. Plots were performed by using GraphPad Prism 8.0.2.

## Results

### TAX increased boar semen quality

As shown in Figure 1A (Study scheme), the adult Duroc boars were fed TAX at 15 mg/kg body weight for 9 weeks. Dietary supplementation of TAX significantly increased sperm motility compared to the control (CON) group (Figure 1B; *p* < 0.05). Meanwhile, TAX tended to increase sperm concentration (Figure 1C; *p =* 0.106). However, there were no differences in the abnormal sperm rate between the TAX and CON groups (Figure 1D). The data suggested that TAX improved semen quality by increasing sperm motility and raising the tendency of sperm concentration.

### TAX increased the protein level related to spermatogenesis

**Figure 2:**
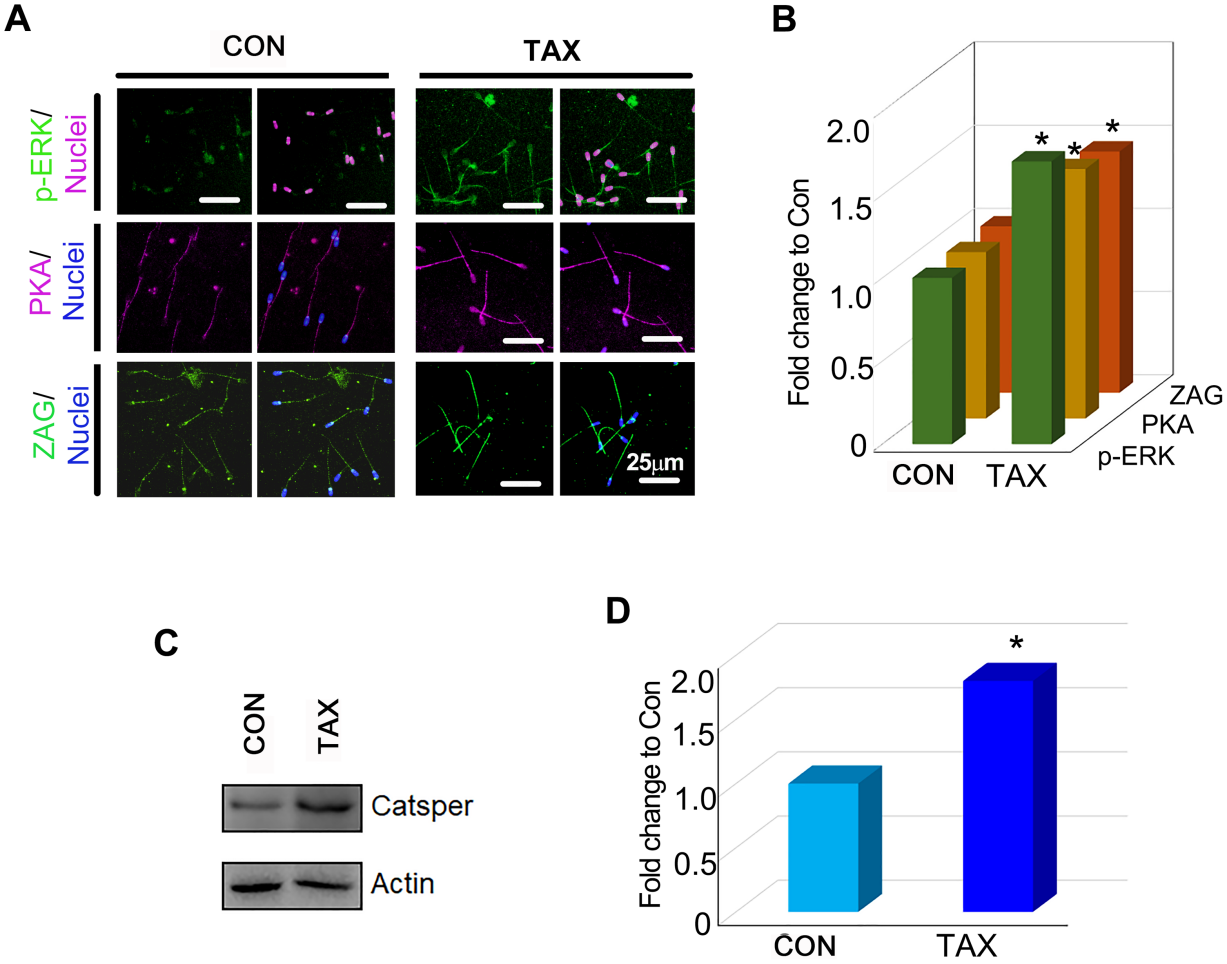
Effects of TAX on the protein expression of important genes related to sperm quality. **(A)** Immunofluorescence staining (IHF) of p-ERK, PKA, and ZAG. **(B)** Quantitative data for IHF staining of p-ERK, PKA, and ZAG (Fold change to CON). **(C)** Western blotting (WB) of Catsper. **(D)** Quantitative data for Catsper staining (Fold change to CON). *p < 0.05.

To understand how TAX improved boar semen quality, the protein levels (p-ERK, PKA, ZAG, and CatSper; Liu et al., 2012; Li et al., 2016; Xu et al., 2018) of important genes for sperm quality were quantified. TAX increased the protein levels of p-ERK, PKA and ZAG significantly compared to the CON group by IHF staining (Figure 2A,B; *p* < 0.05). Then, we used Western Blotting experiments to further confirm the results above (Figures 2C,D; *p* < 0.05). The results indicated that TAX could improve sperm quality by increasing the proteins related to spermatogenesis.

### TAX benefited blood metabolites to improve the semen quality of Duroc boars

**Figure 3:**
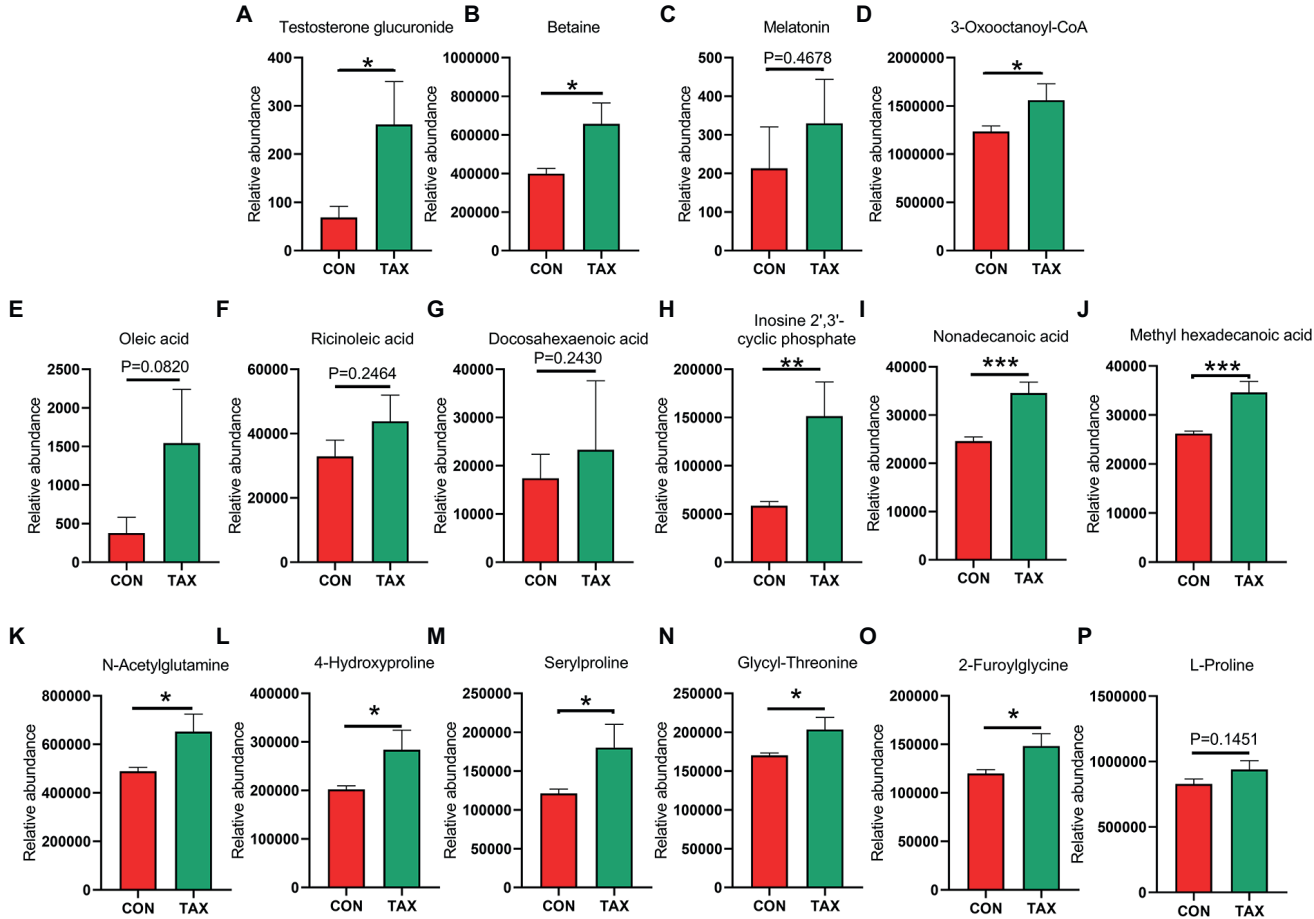
TAX increased blood metabolites. **(A)** Blood testosterone glucuronide level. **(B)** Blood betaine level. **(C)** Blood melatonin level. **(D)** Blood 3-Oxooctanoyl-CoA level. **(E)** Blood Oleic acid level. **(F)** Blood Ricinoleic acid level. **(G)** Blood DHA level. **(H)** Blood Inosine 2′,3′-cyclic phosphatel level. **(I)** Blood Nonadecanoic acid level. **(J)** Blood Methyl hexadecanoic acid level. **(K)** Blood N-Acetylglutamine level. **(L)** Blood 4-Hydroxyproline level. **(M)** Blood Serylproline level. **(N)** Blood Glycyl-Threonine level. **(O)** Blood 2-Furoylglycine level. **(P)** Blood L-Proline level. Data were expressed as the mean ± SEM. The *y*-axis represents the relative amount. The *x*-axis represents the treatments. ^*^p < 0.05. ^**^p < 0.01. ^***^p < 0.001.

**Figure 4:**
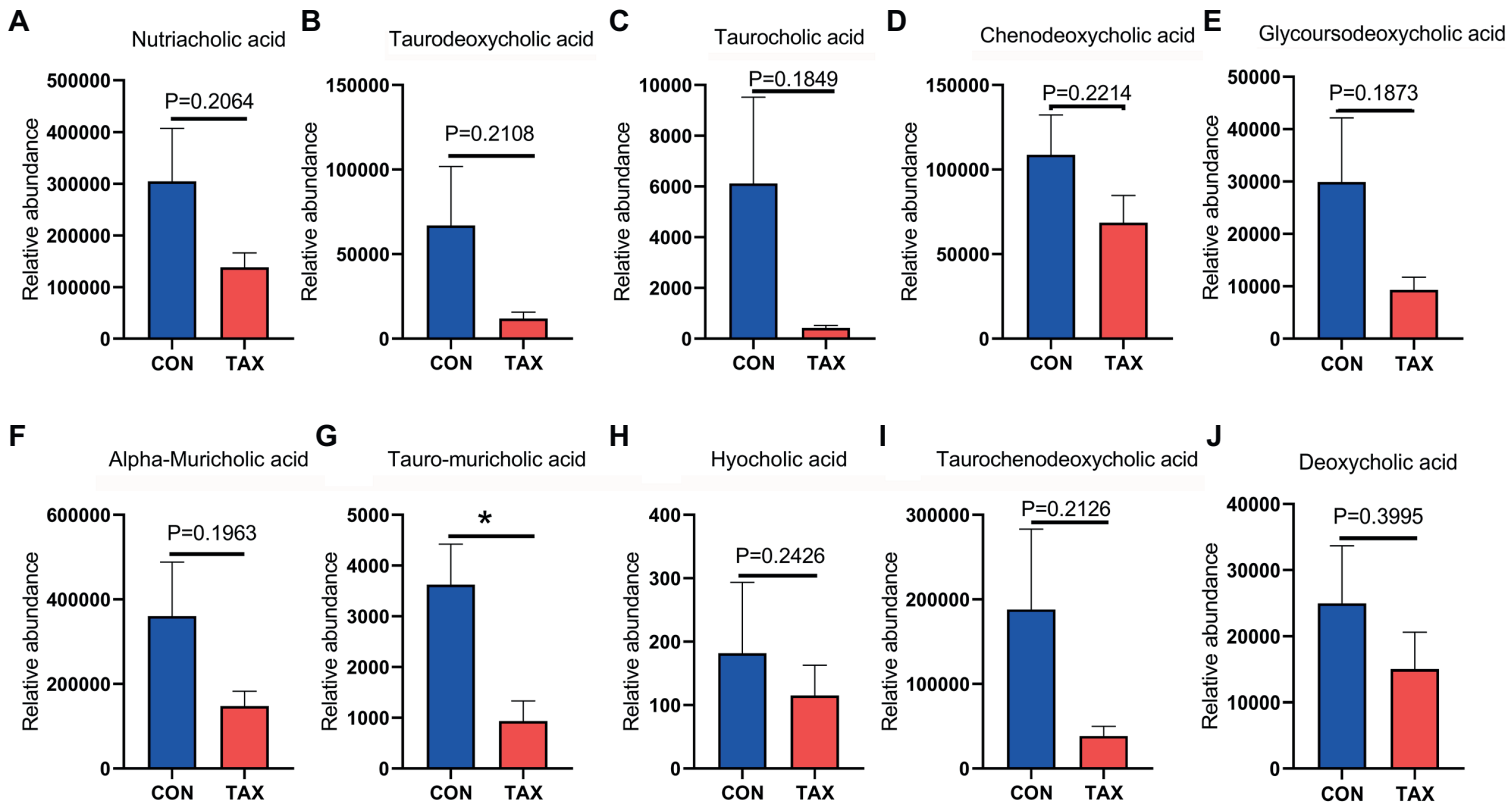
TAX decreased blood bile acids. **(A)** Blood nutriacholic acid level. **(B)** Blood Taurodeoxycholic acid level. **(C)** Blood taurocholic acid level. **(D)** Blood Chenodeoxycholic acid level. **(E)** Blood Glycoursodeoxycholic acid level. **(F)** Blood Alpha-Muricholic acid level. **(G)** Blood Tauro-muricholic acid level. **(H)** Blood Hyocholic acid level. **(I)** Blood Taurochenodeoxycholic acid level. **(J)** Blood Deoxycholic acid level. Data were expressed as the mean ± SEM. The y-axis represents the relative amount. The x-axis represents the treatment. ^*^p < 0.05.

TAX altered the blood metabolites, which were determined by LC/MS analysis (Data File 1). Firstly, TAX increased the level of blood steroid hormone testosterone glucuronide (Figure 3A; *p < 0.05). TAX elevated a batch of blood antioxidants such as Betaine (*p < 0.05), Melatonin (*p* = 0.46), and 3-Oxooctanoyl-CoA (*p < 0.05) compared to the CON group (Figures 3B–D). Meanwhile, we also found that a few fatty acids, including Oleic acid (*p* = 0.082), Ricinoleic acid (*p* = 0.2464), DHA (*p* = 0.243), Inosine cyclic phosphate (**p < 0.01), Nonadecanoic acid (***p < 0.001), and Methyl hexadecanoic acids (***p < 0.001) were increased in TAX group than CON group (Figures 3E–J). Moreover, TAX significantly increased 5 amino acids and derivatives such as N-Acetylglutamine (*p < 0.05), 4-Hydroxyproline (*p < 0.05), Serylproline (*p < 0.05), Glycyl-Threonine (*p < 0.05), and 2-Furoylglycine (*p < 0.05) compared to the CON group (Figures 3K–P). It was very interesting to notice that TAX reduced ten different kinds of bile acids and derivatives compared to the CON group (Figures 4A–J).

### TAX changed the gut microbial composition of boars

**Figure 5:**
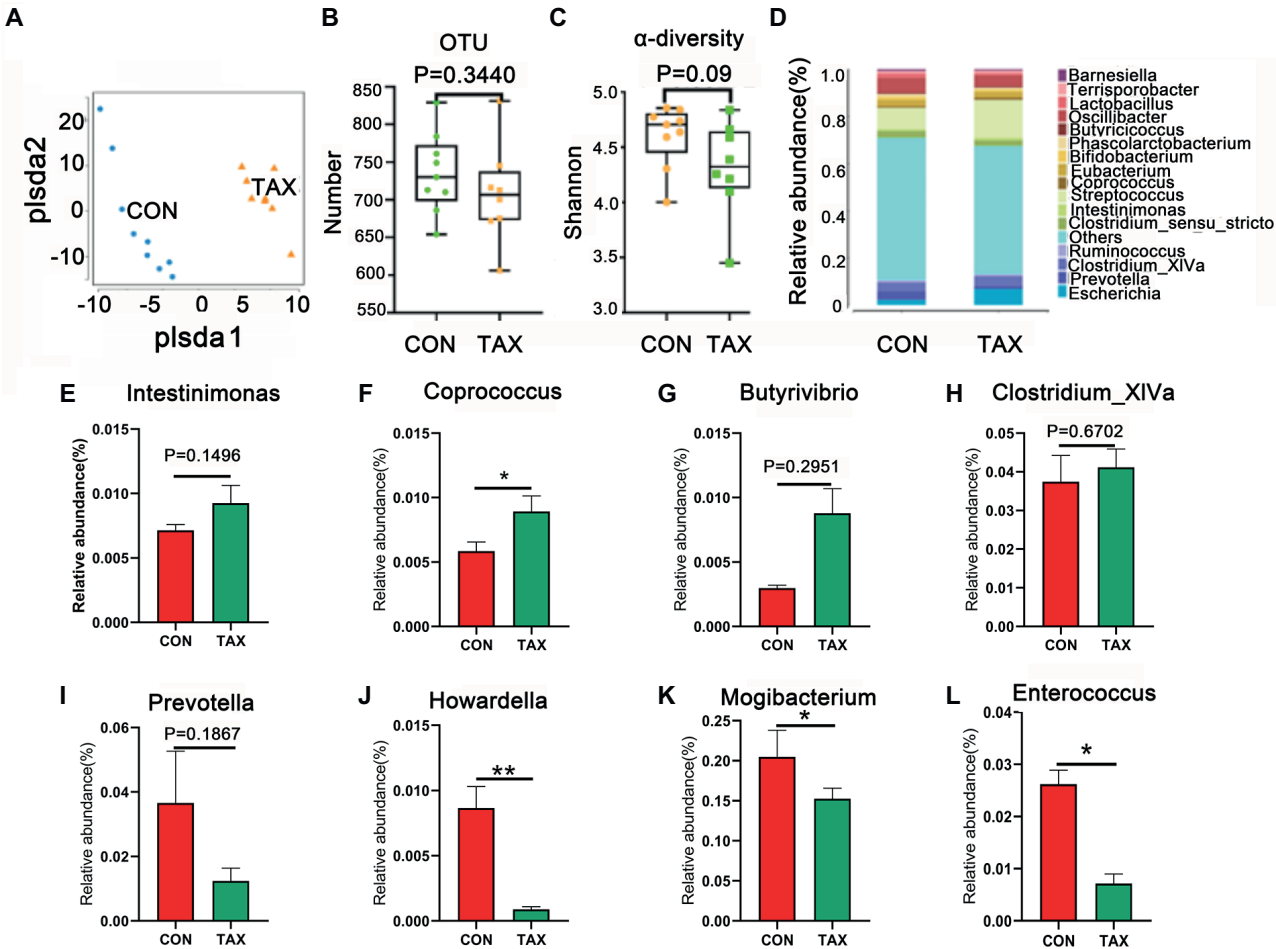
Effects of TAX on the fecal microbial composition. **(A)** The PLS-DA analysis of OUT of fecal microbes. **(B)** The levels of OUTs. **(C)** α-diversity with Shannon. **(D)** The relative amount of microbiota in feces at the genus level. The relative amount of individual microbiota in feces at the genus level **(E–L)**. Data were expressed as the mean ± SEM. *p < 0.05. ^**^p < 0.01.

To search for the beneficial advantages of TAX on gut microbes, fecal microbes were determined. The microbes were different between the TAX and CON groups by PLS-DA analysis (Figure 5A), however, the total OUT and α-diversity were not changed much (Figures 5B,C). TAX increased the abundance of beneficial microbiota such as *Intestinimonas* (*p* = 0.1496), *Coprococcus* (*p < 0.05), *Butyrivibrio* (*p* = 0.2951), and *Clostridium_XlVa* (*p* = 0.6702) at the Genus level (Figures 5E–H). However, the harmful microbes were decreased by TAX such as *Prevotella* (*p* = 0.1867), *Howardella* (**p < 0.01), *Mogibacterium* (*p < 0.05), and *Enterococcus* (*p < 0.05; Figures 5I–L).

### Spearman correlation among fecal microbes, plasma metabolites, and sperm parameters

**Figure 6:**
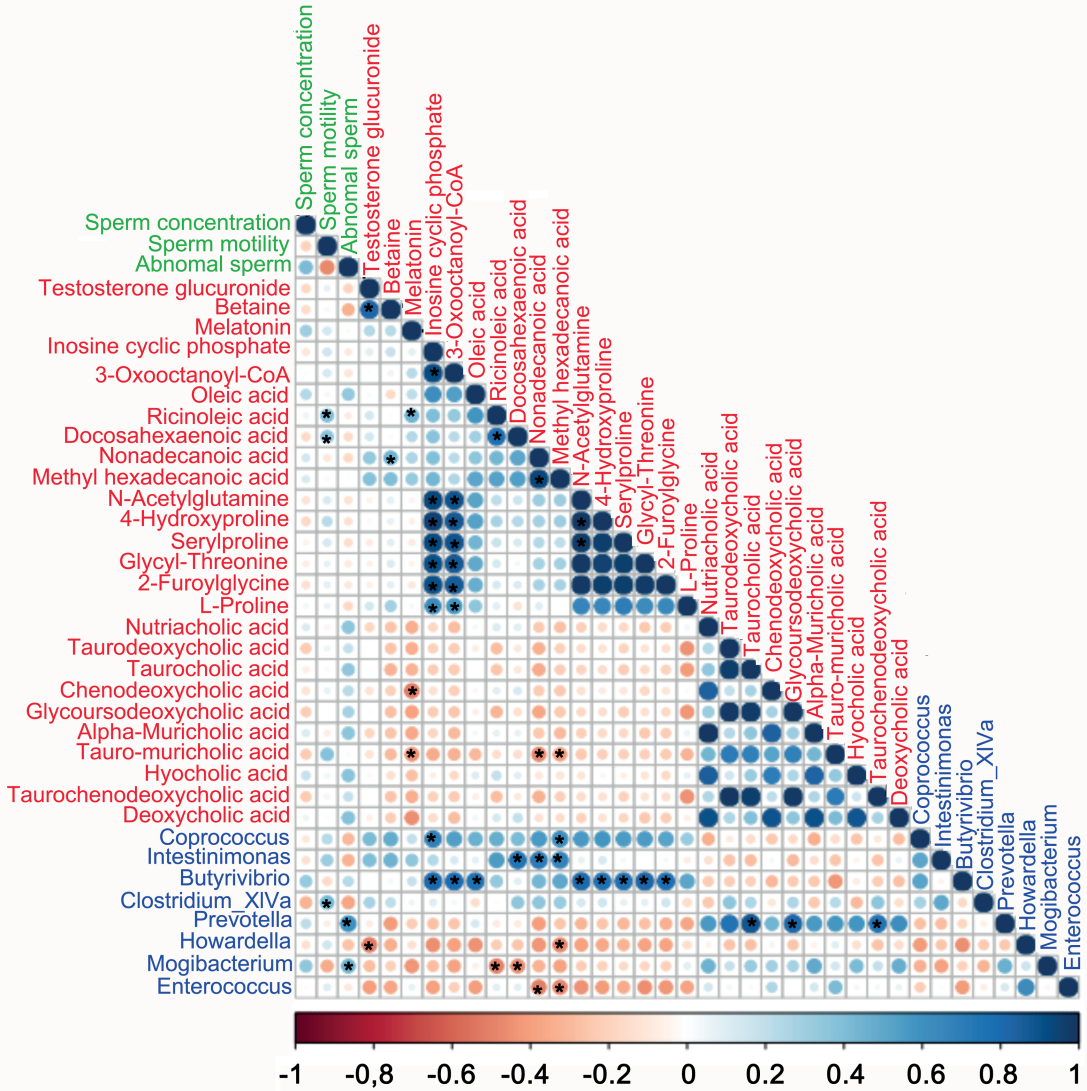
Correlations among fecal microbes, blood metabolites, and semen quality parameters. The color of the circle represents a positive or negative correlation, and the size of the circle represents the strength of the correlation. (large circle = stronger correlation, color green represents semen quality parameters, the color red represents blood metabolites, the color blue represents fecal microbes). *p < 0.05.

Spearman correlation analysis (Figure 6) indicated that the fecal microbes, plasma metabolites, and semen parameters were well correlated. Firstly, the blood metabolites were well correlated with each other. Secondly, there was also a good correlation between blood metabolites and gut microbes. In the TAX group, the elevated beneficial bacteria were positively correlated with amino acids and unsaturated fatty acids, and negatively correlated with bile acids. Conversely, decreased harmful bacteria were negatively correlated with amino acids and unsaturated fatty acids and positively correlated with bile acids. Among them. The beneficial bacteria *Butyrivibrio* was significantly positively correlated with amino acids. The harmful bacteria *Prevotella* was significantly positively correlated with bile acids. In terms of semen quality, unsaturated fatty acids Ricinoleic acid and DHA had a strong positive correlation with sperm motility, while harmful bacteria *Prevotella* were significantly positively correlated with abnormal sperm rate. There was also a trend of positive correlation between bile acids and abnormal sperm.

## Discussion

As a natural product, TAX has multiple biological functions. In recent years, it has been used in many fields, such as anti-oxidation to scavenge free radicals (Li et al., 2017; Gustiene et al., 2019; Lektemur Alpan et al., 2020), anti-obesity (Su et al., 2022), anti-inflammation (Wu et al., 2019), and other areas. In the current research, we found that adding TAX to the basal diet could improve boar semen quality by increasing sperm motility and sperm concentration. It has been reported that adding TAX to the cryopreservation extender can improve the ram sperm quality (Bucak et al., 2020). We also found that TAX increased the levels of some important proteins related to spermatogeneses such as ZAG, PKA, CatSper, and p-ERK. ZAG has been found to increase sperm motility (Qu et al., 2007). Catsper regulates sperm tail calcium entry and sperm hyperactivated motility (Lishko et al., 2012). PKA is related to sperm capacitation in mammalian (Baro Graf et al., 2020). p-ERK was related to sperm concentration and sperm activity (Li et al., 2016). In another study, TAX could rescue di-n-butyl phthalate disrupted testicular development in prenatal rats (Li Z. et al., 2020). Therefore, TAX improves the semen quality of Duroc boars by increasing some protein levels that benefit spermatogenesis.

Intestinal microbes not only regulate host health but also play an important role as a bridge between diet and host. The beneficial effects of TAX on biological systems may be through changing gut microbial composition. It has been reported that TAX improved dysbiosis caused by a high fat diet and regulated the gut microbiota diversity, also decreasing the ratio of *Firmicutes*/*Bacteroidetes*, which inhibit *Proteobacteria* from blooming (Su et al., 2022). Dietary TAX prevented dextran sulfate sodium (DSS)-induced colitis by reducing the abundance of *Bacteroides*, *Clostridium ramosum*, *Clostridium saccharogumia*, *Sphingobacterium multivorum*. Meanwhile, there was an increase in the abundance of *Desulfovibrio* and *Gemmiger formicilis* at the genus level (Hou et al., 2021). TAX ameliorated the aging process by modifying the gut microbes: *Enterorhabdus*, *Clostridium*, *Bifidobacterium*, and *Parvibacter* (Liu et al., 2021b). In our previous study, we found that alginate oligosaccharides (AOS; a natural antioxidant) could increase the “beneficial” bacteria such as *Bacteroidales*, *Lactobacillaceae,* and *Campylobacterales* to improve spermatogenesis and semen quality (Zhao et al., 2020; Zhang P. et al., 2021; Zhang C. et al., 2021). In this study, we found that dietary addition TAX increased the level of *Coprococcus* (butyrate producing; Keshavarzian et al., 2015), *Intestinimonas* (butyrate producing; Afouda et al., 2019), *Butyrivibrio* which is fermented glucose to produce butyric acid, then synthesizes short-chain fatty acids to protect the function of the intestinal epithelium, and some short-chain fatty acids can also be used in spermatogenesis (Moon et al., 2008; Olia Bagheri et al., 2021). On the other hand, TAX decreased the levels of “harmful” bacteria such as *Enterococcus*, *Prevotella*, *Howardella,* and *Mogibacterium*. A study has shown that *Enterococcus* can induce Bacteriospermia in rabbit semen (Duracka et al., 2019). *Prevotella* appeared to exert a negative effect on sperm quality (Farahani et al., 2021). In our investigation, we also found that *Prevotella* was significantly positively correlated with abnormal sperm rate. Therefore, our results were consistent with previous studies. It was interesting to notice that *Howardella* was associated with obesity (Zhu et al., 2021), which was also an important factor affecting semen quality and male infertility (Leisegang et al., 2021). TAX could decrease the abundance of *Mogibacterium* which promoted inflammation and is associated with obesity (Wu et al., 2018; Li Q. et al., 2020). Therefore, dietary supplementation of TAX can improve the semen quality of boars by increasing “beneficial” bacteria and decreasing “harmful” bacteria.

Metabolic regulation plays a crucial role in spermatogenesis (Rato et al., 2012; Al-Asmakh et al., 2014; Dai et al., 2015), and gut microbiota can produce metabolites to modulate systemic metabolome (Hou et al., 2021; Su et al., 2022). It has been reported that TAX could improve the blood metabolites of pigs to prevent oxidative stress (Nekrasov et al., 2021). In current experiments, TAX increased blood testosterone and the derivatives which were essential for maintaining spermatogenesis and boar fertility (Smith and Walker, 2014). Moreover, TAX increased blood antioxidant molecules melatonin (increased trend) and betaine. Melatonin has been reported to improve spermatogenesis *via* the alleviation of oxidative stress and DNA damage (Pang et al., 2017; Xu et al., 2020). Betaine could improve sperm quality and ameliorate oxidative damage in testis (Elsheikh et al., 2020). Furthermore, TAX increased (n-3) polyunsaturated fatty acids in the blood such as docosahexaenoic acid (DHA) which was very crucial for spermatogenesis and sperm quality (Hale et al., 2019; Bunay et al., 2021). In addition, TAX elevated some essential amino acids such as proline, which is important for spermatogenesis and semen quality (Dawra et al., 2015; Dong et al., 2016). However, TAX reduced blood bile acid derivatives, which have been reported to induce oxidative stress (Bomzon et al., 1997). Various studies have reported that bile acids can result in oxidative stress by promoting the production of oxygen free radicals from mitochondria (Bomzon et al., 1997). Moreover, bile acids contributed to infertility by activating farnesoid X receptor and G-protein-coupled bile acid receptor expressed in sperm, which then affected glucose and lipid metabolism and led to abnormal sperm (Baptissart et al., 2014; Malivindi et al., 2018). Our data were consistent with the previous studies described above. Therefore, dietary supplementation of TAX can improve the blood metabolites of boars to keep them healthy. However, there are some limitations to this study, meaning that the underlying mechanism of TAX improved sperm motility or concentration was not fully revealed. In our previrous research, we found that Hydroxytyrosol which is a kind of antioxidant benefits the semen quality of Duroc boar through improving gut microbes and blood metabolites (Han et al., 2021). In our current research, we also found that the blood metabolites and gut microbes have a good correlation with each other, so the improvement of boar semen quality by TAX was mainly mediated by both blood metabolites and gut microbes.

The present study indicated that TAX improves the semen quality of boars by ameliorating gut microbiota and blood metabolome. Our study confirms that TAX may be a good feed additive for improving the semen quality of boars, increasing the conception rate and litter size of sows to meet demands for pork consumption.

## Data availability statement

The datasets presented in this study can be found in online repositories. The names of the repository/repositories and accession number(s) can be found in the article/Supplementary material.

## Ethics statement

The animal study was reviewed and approved by Animal Care and Use Committee of the Institute of Animal Sciences of Chinese Academy of Agricultural Sciences (IAS2021-67).

## Author contributions

YZ, HZ, and YG designed the experiment. YZ, LC, HH, RZ, BX, LL, HS, JT, XC, and YG conducted the experiment and analyzed the data. MS, HZ, and YZ wrote and edited the manuscript. All authors contributed to the article and approved the submitted version.

## Funding

This research was supported by funding from China Academy of Agriculture Sciences, the Agricultural Science and Technology Innovation Program (CAAS-ZDRW202006-02, ASTIPIAS07), and the State Key Laboratory of Animal Nutrition (2004DA125184G2102).

## Conflict of interest

XC was employed by the company Yinuo Biopharmaceutical Co., Ltd.

The remaining authors declare that the research was conducted in the absence of any commercial or financial relationships that could be construed as a potential conflict of interest.

## Publisher’s note

All claims expressed in this article are solely those of the authors and do not necessarily represent those of their affiliated organizations, or those of the publisher, the editors and the reviewers. Any product that may be evaluated in this article, or claim that may be made by its manufacturer, is not guaranteed or endorsed by the publisher.
