# Corrigendum: Taxifolin increased semen quality of Duroc boars by improving gut microbes and blood metabolites

**DOI:** 10.3389/fmicb.2025.1566192

**Published:** 2025-02-07

**Authors:** Yexun Zhou, Liang Chen, Hui Han, Bohui Xiong, Ruqing Zhong, Yue Jiang, Lei Liu, Haiqing Sun, Jiajian Tan, Xiaowei Cheng, Martine Schroyen, Yang Gao, Yong Zhao, Hongfu Zhang

**Affiliations:** ^1^State Key Laboratory of Animal Nutrition, Institute of Animal Sciences, Chinese Academy of Agricultural Sciences, Beijing, China; ^2^Precision Livestock and Nutrition Unit, Gembloux Agro-Bio Tech, University of Liège, Gembloux, Belgium; ^3^YangXiang Joint Stock Company, Guigang, China; ^4^Yinuo Biopharmaceutical Co., Ltd, Harbin, China; ^5^College of Life Science, Baicheng Normal University, Baicheng, Jilin, China

**Keywords:** Taxifolin, semen quality, blood metabolite, gut microbiota, boar

In the published article, there was an error in Figure 2A as published. The photos for CON group for PKA/Nuclei and ZAG/Nuclei are vague and are similar to the photos in our another article.

The corrected Figure 2A and its caption appear below.

**Figure 2:**
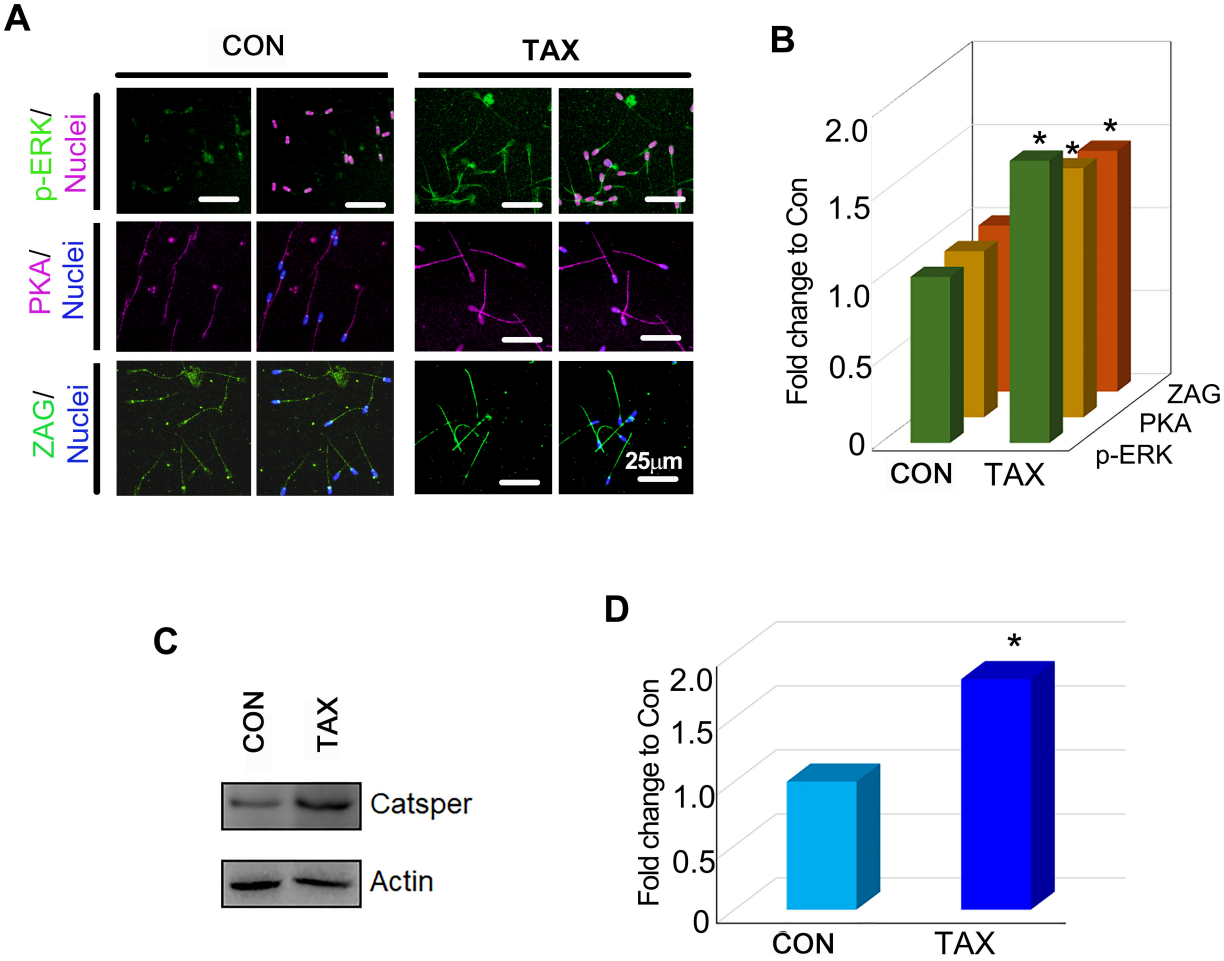
Effects of TAX on the protein expression of important genes related to sperm quality. **(A)** Immunofluorescence staining (IHF) of p-ERK, PKA, and ZAG. **(B)** Quantitative data for IHF staining of p-ERK, PKA, and ZAG (Fold change to CON). **(C)** Western blotting (WB) of Catsper. **(D)** Quantitative data for Catsper staining (Fold change to CON). *p < 0.05.

The authors apologize for this error and state that this does not change the scientific conclusions of the article in any way. The original article has been updated.

