# Supplementary material for: Taxifolin increased semen quality of Duroc boars by improving gut microbes and blood metabolites
Source: Front Microbiol. 2022 Oct 14;13:1020628. doi: 10.3389/fmicb.2022.1020628 (PMC9614168; doi:10.3389/fmicb.2022.1020628)
Supplement: Supplementary file 3 [file Table_4.DOCX]

**Table S2.** Primary antibody information

| **Gene symbol** | **Name** | **Cat. #** | **Predicted size** | **Source (Animal)** | **Company** |
| --- | --- | --- | --- | --- | --- |
| Actin | actin | Ab3280 | 42kDa | Rabbit (polyclonal) | Abcam |
| PKA | cAMP dependent protein kinase alpha catalytic subunit | bs-0520R | 40kd | Rabbit (polyclonal) | Beijing Biosynthesis Biotechnology CO. |
| P-ERK | phospho-Erk1 (Thr202 + Tyr204) | bs-1645R | 43kDa | Rabbit (polyclonal) | Beijing Biosynthesis Biotechnology CO. |
| ZAG | Zinc Alpha 2 Glycoprotein | bs-19382R | 32kDa | Rabbit | Beijing Biosynthesis Biotechnology CO. |
| CATSPER | CATSPER | bs-23326R | 90kDa | Rabbit | Beijing Biosynthesis Biotechnology CO. |
